# The effect of lymph node dissection on oncological outcomes in contemporary robotic salvage radical prostatectomy patients: a junior ERUS/YAU collaborative study

**DOI:** 10.1007/s00345-025-05586-5

**Published:** 2025-05-09

**Authors:** Mike Wenzel, Christoph Würnschimmel, Marcio Covas Moschovas, Arjun Nathan, Christian Wagner, Giorgio Calleris, Fabrizio Di Maida, Juan Gomez Rivas, Carlo Andrea Bravi, Ruben De Groote, Federico Piramide, Filippo Turri, Keith Kowalczyk, Gopal Sharma, Iulia Andras, Edward Lambert, Nikolaos Liakos, Danny Darlington, Marco Paciotti, Gabriele Sorce, Philipp Mandel, Antonio Galfano, Giancarlo Marra, Senthil Nathan, Paolo Dell’Oglio, Alexandre Mottrie, Felix K. H. Chun, Vipul Patel, Alberto Breda, Alessandro Larcher

**Affiliations:** 1 Department of Urology, Goethe University Hospital Frankfurt, Frankfurt am Main, Germany; 2 Department of Urology, Luzerner Kantonsspital, Lucerne, Switzerland; 3 AdventHealth Global Robotics Institute, Celebration, FL USA; 4 University College London Hospitals NHS Foundation Trust, London, UK; 5 Prostate Center Northwest, Department of Urology, Pediatric Urology and Uro Oncology, St. Antonius-Hospital, Gronau, Germany; 6 Department of Surgical Sciences, Urology Clinic, University of Turin and Città della Salute e della Scienza, Turin, Italy; 7 Department of Experimental and Clinical Medicine, Unit of Oncologic Minimally-Invasive Urology and Andrology, University of Florence, Careggi Hospital, Florence, Italy; 8 Department of Urology, Hospital Clínico San Carlos, Madrid, Spain; 9 Department of Urology, The Royal Marsden NHS Foundation Trust, London, UK; 10 Department of Urology, Onze-Lieve-Vrouwziekenhuis Hospital, Aalst, Belgium; 11 ORSI Academy, Ghent, Belgium; 12 Division of Urology, Department of Oncology, San Luigi Gonzaga Hospital, University of Turin, Turin, Italy; 13 Department of Urology, ASST Santi Paolo e Carlo, University of Milan, Milan, Italy; 14 Department of Urology, District of Columbia, MedStar Georgetown University Hospital, Washington, USA; 15 Department of Urologic Oncology, Medanta The Medicity, Gurgaon, India; 16 Department of Urology, Iuliu Hatieganu University of Medicine and Pharmacy, Cluj- Napoca, Romania; 17 Department of Urology, Ghent University Hospital, Ghent, Belgium; 18 Department of Urology, Faculty of Medicine, Medical Centre of the University of Freiburg, Freiburg, Germany; 19 Department of Urology, Stokes Centre for Urology, Royal Surrey County Hospital, Egerton Road, Guildford, GU2 7XX UK; 20 Department of Urology, Humanitas Research Hospital- IRCCS, Rozzano, Italy; 21 Department of Urology, IRCCS San Raffaele Hospital, Milan, Italy; 22 ASST Grande Ospedale Metropolitano Niguarda Urology Department, Milan, Italy; 23 Department of Urology, Fundació Puigvert, Universitat Autònoma de Barcelona, Barcelona, Spain

**Keywords:** SRARP, Recurrent prostate cancer, BCR, MFS, Brachytherapy

## Abstract

**Purpose:**

Surgical features associated with better cancer-control outcomes are under investigation for salvage radical prostatectomy patients undergoing robotic approaches.

**Methods:**

The Junior ERUS/Young Academic Urologist Working Group in Robotics in Urology conducted a multicentric project to investigate the effect of lymph node dissection (LND) and pN stage on biochemical recurrence-free (BCR), metastases-free (MFS) and overall survival (OS) outcomes in 444 robotic salvage radical prostatectomy (s-RARP) patients.

**Results:**

Of all patients, 63% underwent LND with a median of eight removed lymph nodes. Patients without LND more frequently underwent initial focal therapy (60% vs. 37%) and harbored higher pathological Gleason score 8–10 (both *p* ≤ 0.02). In BCR analyses, no differences were observed between patients with vs. without LND with 24-months BCR-free survival rates of 74.8% vs. 73.6%. In OS analyses, better OS for LND patients (HR: 0,39, *p* = 0.049) was observed with 60-months OS rates of 81.3% vs. 92.1% for no LND vs. LND. Of LND patients. 16% harbored pN1 stage, which was associated with worse BCR-free survival (HR: 2.0,*p* = 0.025) with 60-months BCR-free survival rates of 63% for pN0 vs. 49.6% for pN1. In MFS analyses, no difference between both groups were observed. In OS analyses, the observed differences did not reach statistical significance with 60-months OS rates of 93.4% for pN0 vs. 84.7% for pN1.

**Conclusion:**

Rates of LND are relatively low in contemporary s-RARP patients and are more frequently performed in patients with worse characteristics. However, LND may be associated with better OS, while pN1 patients are associated with worse cancer-control outcomes.

## Introduction

Outcomes of salvage treatment options such as salvage radical prostatectomy for relapsing prostate cancer after failure of the primary curative therapy such as radiation therapy or focal therapy are under current scientific investigation [1–3]. Due to the poor functional outcomes after salvage radical prostatectomy, EAU guidelines recommend this salvage procedure in highly selected patients with favorable tumor characteristics in highly experienced centers only, which makes salvage radical prostatectomies nowadays a relatively rarely performed procedure [4–7].

For primary radical prostatectomy, as well as salvage radical prostatectomy, a robotic approach has been established as surgical gold standard approach and seems to be associated with better perioperative and functional outcomes [8–10]. However, most studies focusing on robotic salvage radical prostatectomy are either case reports or mixed-cohorts including patients with open and robotic approaches or samples from an inclusion period of over two decades in order to achieve sufficient sample sizes [11–16]. Therefore, little is known about the effect of surgical techniques such as the impact of lymph node dissection (LND) during robotic salvage radical prostatectomy on cancer-control outcomes, and no guidelines explicitly recommend performing it.

To address this void, we conducted a multi-center study within high volume robotic prostate cancer centers across the world and within the Junior ERUS/Young Academic Urologist Working Group on Robotics in Urology. We hypothesized that LND rates in contemporary patients treated with robotic salvage radical prostatectomy are high and important differences may exist according to oncological outcomes such as biochemical recurrence (BCR)-free survival, metastasis-free survival (MFS) or overall survival (OS) rates between patients with vs. without LND.

## Materials and methods

### Study population

With approval from the local ethics committee at the primary investigator’s center (approval number: SUG-5-2018) and in accordance with the Declaration of Helsinki, the Junior ERUS/Young Academic Urologist Working Group on Robotics in Urology conducted a multicenter study on patients who underwent robotic salvage radical prostatectomy between 2008 and 2023 after initial prostate cancer treatment. This study involved 13 centers worldwide and utilized a multicenter database. Patients with metastatic disease or castration-resistant prostate cancer were excluded. Patients needed to be confirmed with recurrent prostate cancer in biopsy prior to robotic salvage radical prostatectomy. These criteria yielded 444 robotic salvage radical prostatectomy patients for further analyses.

### Data collection and oncological outcomes

All retrospectively collected data, including baseline patient and tumor characteristics, as well as surgical and oncological outcomes of patients undergoing robotic salvage radical prostatectomy, were anonymously sampled. Biochemical recurrence (BCR) was defined according to EAU criteria as a PSA increase to ≥ 0.2 ng/ml after robotic salvage radical prostatectomy [7]. Patients with persistent PSA levels after the procedure were excluded from the BCR analyses. MFS was defined as the time from robotic salvage radical prostatectomy until the first occurrence of non-regional metastasis, while OS was defined as the time from robotic salvage radical prostatectomy until death from any cause.

### Statistical analysis

Descriptive statistics included frequencies and proportions for categorical used variables. Medians and interquartile ranges (IQR) were reported for all applied continuously-coded variables. The Chi-square test was used to test for statistical significance in proportions’ differences. In addition, the Fisher’s exact test and Wilcoxon rank-sum test examined the statistical significance of distributions’ differences. Postoperative complications were recorded according to Clavien Dindo classification.

For oncological BCR-, MFS- and OS outcome analyses, Kaplan Meier curves analyses in robotic salvage radical prostatectomy patients were stratified according to the performance of LND vs. no LND. Moreover, patients were stratified according to pathological nodal status (pN0 vs. pN1 and pN0 vs. pN1 vs. pNx).

In all sets of analyses, univariable, as well as multivariable Cox regression models were applied in order to adjust for potential confounding baseline and pathological tumor characteristics. Multivariable adjustment was performed for age at surgery, primary treatment (focal therapy vs. radiation therapy), positive surgical margins (PSM), PSA prior to surgery, pathological stage and pathological Gleason score, if not variable of outcome interest. For some MFS or OS analyses due to few events and to prevent overfitting the model, no additional adjustments could be made. All tests were two sided with a level of significance set at *p* < 0.05. R software environment for statistical computing and graphics (version 3.4.3) was used for all analyses.

## Results

### Descriptive analyses

Overall, 444 robotic salvage radical prostatectomy patients qualified for analyses (Table 1) with a median follow up of 24 months (IQR: 10–48 months). Median time interval between primary cancer treatment and robotic salvage radical prostatectomy was 48 months (IQR: 24–77) at a median age at surgery of 68 years (IQR: 63–72) with a PSA of 4.5 ng/ml (IQR: 2.7-7.5ng/ml).

**Table 1:** Characteristics of 444 robotic salvage radical prostatectomy (sRP) patients stratified lymph node dissection (LND) performed vs. not performed

Characteristic	*N*	Overall, *N* = 444^*1*^	No LND, *N* = 166 (37%)^*1*^	LND, *N* = 278 (63%)^*1*^	*p*-value^2^
Month between initial PCa and sRP	367	48 (24, 77)	41 (24, 64)	52 (24, 87)	0.048
Age initial PCa	183	64 (59, 69)	65 (60, 68)	63 (59, 69)	0.7
Age sRP	377	68 (63, 72)	69 (64, 72)	67 (63, 72)	0.078
CCI initial PCa	201	0 (0, 1)	0 (0, 1)	1 (0, 5)	< 0.001
CCI sRP	206	3 (2, 4)	4 (3, 5)	3 (2, 4)	0.028
PSA initial PCa	121	7 (6, 10)	6 (5, 8)	7 (6, 10)	0.3
PSA prior sRP	396	4.5 (2.7, 7.5)	5.2 (3.5, 7.7)	3.8 (2.4, 7.4)	0.005
Positive cores initial PCa	69	4 (2, 5)	3 (2, 4)	4 (2, 6)	0.2
Positive cores prior sRP	98	4.0 (2.0, 6.0)	3.0 (2.0, 4.5)	4.0 (2.0, 7.0)	0.2
Tumor infiltration initial PCa, %	37	50 (20, 80)	11 (10, 12)	50 (24, 80)	0.004
Tumor infiltration prior sRP, %	74	50 (19, 77)	35 (15, 50)	50 (20, 80)	0.091
LND: number of nodes	271			8 (4, 13)	
EBL	407	100 (100, 250)	180 (100, 250)	100 (100, 200)	0.010
OR time	439	145 (120, 180)	155 (120, 180)	140 (119, 180)	0.031
cT prior sRP ≥ 3–4	364	89 (24%)	60 (38%)	29 (14%)	< 0.001
GS at PCa diagnosis	117				0.009
6		54 (46%)	14 (74%)	40 (41%)	
7		46 (39%)	2 (11%)	44 (45%)	
8–10		17 (15%)	3 (16%)	14 (14%)	
Gleason score at sRP	415				< 0.001
6		58 (14%)	14 (8.5%)	44 (18%)	
7		235 (57%)	112 (68%)	123 (49%)	
8–10		122 (29%)	39 (24%)	83 (33%)	
Primary PCa therapy	439				< 0.001
Focal therapy		201 (46%)	100 (60%)	101 (37%)	
Radiation therapy		238 (54%)	66 (40%)	172 (63%)	
Nerve sparing	443	253 (57%)	64 (39%)	189 (68%)	< 0.001
pT3-4	439	248 (56%)	88 (53%)	160 (59%)	0.3
pN1	418			44 (16%)	
If pN1: Number of positive nodes	44			1 (1, 2)	
PSM	440	135 (31%)	57 (34%)	78 (28%)	0.2
Pathological Gleason sRP	250				0.022
6–7		114 (46%)	18 (32%)	96 (49%)	
8–10		136 (54%)	38 (68%)	98 (51%)	
Complications CD grade	66				0.4
1		43 (65%)	9 (64%)	34 (65%)	
2		12 (18%)	5 (36%)	7 (13%)	
3		5 (7.6%)	0 (0%)	5 (9.6%)	
3a		3 (4.5%)	0 (0%)	3 (5.8%)	
3b		2 (3.0%)	0 (0%)	2 (3.8%)	
4a		1 (1.5%)	0 (0%)	1 (1.9%)	
PSA Persistence	438	41 (9.4%)	11 (6.7%)	30 (11%)	0.13

^*1*^ Median (IQR); n (%)

^*2*^ Wilcoxon rank-sum test; Fisher’s exact test; Pearson’s Chi-square test

*Abbreviations* PCa: Prostate cancer, CCI: Charlson Comorbidity Index, PSA: Prostate-specific antigen, ADT: Androgen deprivation therapy, EBL: Estimated blood loss, OR: Operating room, ECOG: Eastern Cooperative Oncology Group, GS: Gleason Score, PSM: Positive surgical margin, CD: Clavien-Dindo

### Characteristics of LND vs. no LND

Overall, 63% (*n* = 278) of included robotic salvage radical prostatectomy patients underwent LND with median of 8 removed lymph nodes (IQR: 4–13, Table 1). Patients who received LND exhibited a longer time interval between initial prostate cancer treatment and salvage radical prostatectomy (52 vs. 41 months, *p* = 0.048). Moreover, LND patients more frequently harbored unfavorable initial prostate cancer and baseline characteristics such as percentage of tumor infiltration in biopsy cores (50 vs. 11%) or Gleason score 7 at diagnosis (45% vs. 11%) or Gleason score 8–10 prior to salvage surgery (33% vs. 24%), relative to patients who did not receive LND. Additionally, PSA level prior to robotic salvage radical prostatectomy (5.2 vs. 3.8 ng/ml) was higher, similarly as blood loss or surgical time in no LND vs. LND patients (all *p* ≤ 0.03).

Patients who did not receive LND more frequently underwent initial focal therapy (60% vs. 37%) and harbored higher pathological Gleason score 8–10 (68% vs. 51%, both *p* ≤ 0.02). No differences in postsurgical complications were observed (*p* = 0.4) between both groups. PSA persistence after robotic salvage radical prostatectomy was 11% for LND vs. 6.7% for no LND patients (0.13).

### Cancer-control outcomes for LND

**Fig. 1:**
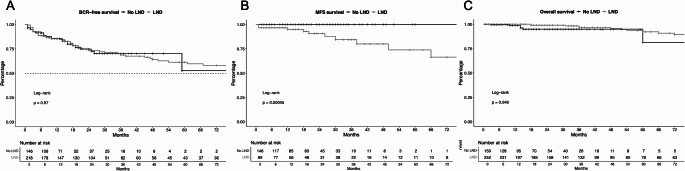
Kaplan Meier curves depicting the effect of lymph node dissection on oncological outcomes such as biochemical-recurrence (BCR, **A**), metastasis-free survival (MFS, **B**) and overall survival (OS, **C**)

In BCR analyses, no difference was observed between patients with vs. without LND (Fig. 1A, *p* = 1.0) with 24- and 60-months BCR-free survival rates of 74.8% and 52.7% vs. 73.6% and 61.3% no LND vs. LND performed at robotic salvage radical prostatectomy. Conversely, patients with LND harbored worse MFS (no HR due to no events for no LND group) with corresponding 24- and 60-months MFS rates of 0% and 0% vs. 90.7% and 73.9% for no LND vs. LND (*p* < 0.01, Fig. 1B). In OS analyses, significant differences between both groups were observed with better OS for LND patients (HR: 0,39, *p* = 0.049, Fig. 1C) and 24- and 60-months OS rates of 94.9% and 81.3% vs. 98.9% and 92.1% for no LND vs. LND. However, after multivariable adjustment, no difference was observed in OS analyses (HR: 0.23, *p* = 0.08).

### Cancer-control outcomes for pN stage

**Fig. 2:**
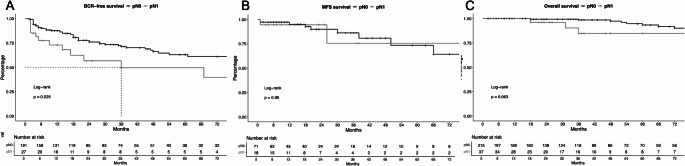
Kaplan Meier curves depicting the effect of patients undergoing lymph node dissection regarding pN stage on oncological outcomes such as biochemical-recurrence (BCR, **A**), metastasis-free survival (MFS, **B**) and overall survival (OS, **C**)

For patients with LND, stratification according to pN stage showed significant worse BCR-free survival for patients with pN1 (HR: 2.0, *p* = 0.025, Fig. 2A) with 24- and 60-months BCR-free survival rates of 76.0% and 63% for pN0 and 56.7% and 49.6% for pN1 salvage robotic radical prostatectomy patients in univariable analyses. However, after multivariable adjustment, no significant difference remained. In MFS analyses, no difference between both groups were observed (Fig. 2B), with 24- and 60-months MFS rates of 89.9% and 73.4% for pN0 vs. 94.4% and 75.6% for pN1. In OS analyses, the observed differences did not reach statistical significance (*p* = 0.063, Fig. 2C) with 24- and 60-months OS rates of 99.4% and 93.4% for pN0 vs. 96.0% and 84.7% for pN1 salvage robotic radical prostatectomy patients.

**Fig. 3:**
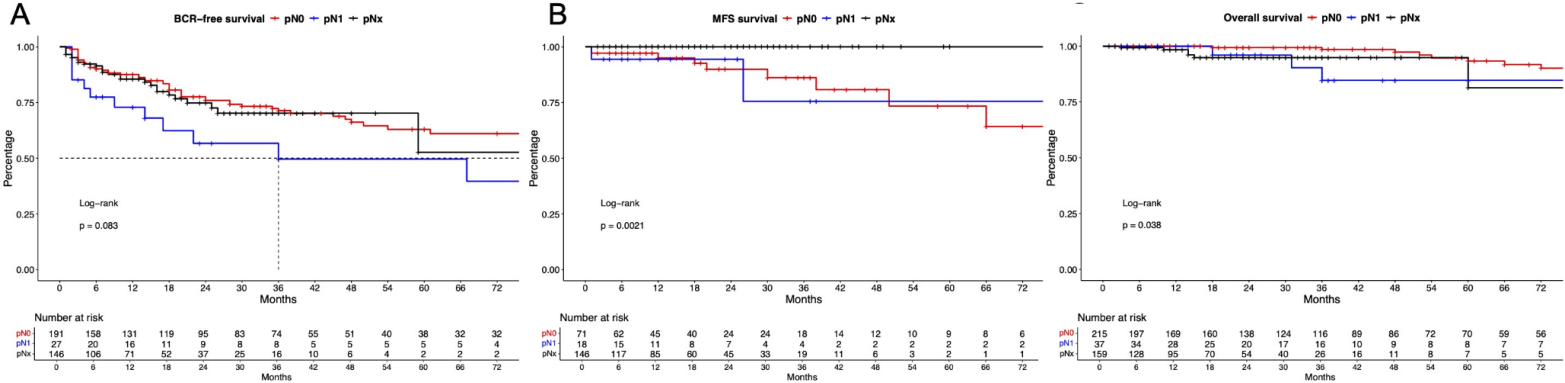
Kaplan Meier curves depicting the effect of pN stage (pN0 vs. pN1 vs. pNx) after robotic salvage radical prostatectomy on oncological outcomes such as biochemical-recurrence (BCR, **A**), metastasis-free survival (MFS, **B**) and overall survival (OS, **C**)

Incorporating pNx stage into pN stage analyses are displayed in Fig. 3. Here, pNx patients were comparable to pN0 patients regarding BCR and OS rates and harbored significant better MFS outcomes (no event for pNx).

## Discussion

We conducted a multi-center study within high volume robotic prostate cancer centers across the world and within the Junior ERUS/Young Academic Urologist Working Group on Robotics in Urology to examine the effect of LND during robotic salvage radical prostatectomy on oncological outcomes such as BCR-free survival, MFS and OS. We hypothesized that important characteristics exist to distinguish contemporary treated patients regarding prognosis in clinical practice. We arrived at several important observations, considering that the current literature lacks orientations to surgeons regarding the benefits, best candidates, and LND templates during robotic salvage prostatectomy.

First, we made important observations regarding characteristics of LND vs. no LND salvage robotic radical prostatectomy patients. For example, only 63% of included robotic salvage radical prostatectomy patients underwent LND. Moreover, the number was even lower for patients initially undergoing focal therapy. This number seems low for high volume surgical centers of prostate cancer care in a salvage setting. However, it may have two explanations: Avoiding side effects such as lymphoceles or the radiation-exposed pelvic tissue which caused an extremely difficult surgical anatomy (only true for RT patients but also seen in OR time analyses). Moreover, the median number of removed lymph nodes were eight, with a pN1 rate of 16%. Additionally, patients undergoing LND harbored worse prostate cancer characteristics at initial prostate cancer diagnosis, as well as at relapsing prostate cancer diagnosis, such as tumor infiltration in biopsy cores or Gleason score 8–10 rates prior to salvage surgery, relative to patients without LND. These findings may lead to selection in those patients to perform LND in order to provide optimal oncological treatment in the setting of salvage robotic radical prostatectomy, which may was not the cause in some focal therapy patients based on physicians’ choice. However, these worse baseline cancer characteristics in addition with the significantly higher rates of pathological Gleason score 8–10 in LND patients may have translated into the observed higher rate of PSA persistence and worse MFS rates in patients undergoing LND. However, in OS analyses, patients who received LND harbored better prognosis regarding OS than patients not undergoing LND. Nonetheless, this effect could not be seen after further multivariable adjustment. Rates of LND in previously published cohorts of salvage radical prostatectomy cohorts widely range between 28 and 85% [17, 18]. However, one more historical and another recently published SEER database report, also found a significant predictive effect on survival in salvage radical prostatectomy undergoing LND with a median number of removed lymph nodes of six in the more contemporary cohort [17, 19]. Conversely, in the previously published multicenter cohort by Preisser et al. a median number of removed lymph nodes were substantially higher with 13 removed lymph nodes [18]. However, similar to our findings, Preisser et al. also did not report a BCR benefit in patients undergoing LND at salvage radical prostatectomy.

The 16% pN1 rate in patients in the current cohort is also comparable regarding to previously published literature. Within one more historical and one more contemporary review regarding salvage radical prostatectomy, rates of lymph node involvement ranged from 5 to 30% [2, 13]. Similar to our findings, Preisser et al. also demonstrated that salvage radical prostatectomy patients with pN1 status exhibited worse BCR and OS [18]. Within our study, only worse BCR outcomes could be investigated as statistically significant, while OS tended to be influenced by lymph node involvement, however did not reach statistical significance in univariable models (*p* = 0.06), possibly due to low number of events and shorter follow up period or limited sample size. Finally, when pNx patients were incorporated, BCR- and OS-outcomes were mostly similar to pN0 patients, which is also consistent to the findings by Preisser et al. [18].

Additionally, to the given retrospective design of our study, further limitation in the interpretation of our study should be acknowledged such as missing data for some possibly confounding variables, missing data on quality of life or functional outcomes after robotic salvage radical prostatectomy. Some of missing pathological data may be partly explained by the destructive effect of the initial radiation therapy. Furthermore, differences in data sampling and follow up periods may limit outcome analyses and reaching statistical significance, similarly as differences in surgical techniques or staging modalities [14]. Finally, staging modalities such as PSMA-PET/CT scan may have impacted the decision on LND performance.

Taken together, our international multicenter YAU collaboration study could demonstrate LND is not routinely performed in contemporary treated salvage robotic radical prostatectomy patients, even in high-volume centers. However, our study could also demonstrate that patients may benefit from LND regarding OS. Finally, our study could demonstrate that 16% of salvage robotic radical prostatectomy patients harbor pathological lymph node involvement, which represents a cohort with worse cancer-control outcomes. Prospective studies should be designed to address the benefits, template extension, and which candidates are the best to undergo LND in Salvage RP.

## Data Availability

No datasets were generated or analysed during the current study.

## Declarations

### Data sharing

Data are available for bona fide researchers who request it from the authors.
